# Turmeric-Associated Drug-Induced Liver Injury

**DOI:** 10.14309/crj.0000000000000941

**Published:** 2022-12-26

**Authors:** Shawnalyn W. Sunagawa, Conor Houlihan, Brandon Reynolds, Sara Kjerengtroen, Daryl J. Murry, Nathalie Khoury

**Affiliations:** 1 Department of Pharmaceutical and Nutrition Care, Nebraska Medicine, Omaha, NE; 2 Department of Internal Medicine, University of Nebraska Medical Center, Omaha, NE; 3 Department of Pharmacy Practice and Science, College of Pharmacy, University of Nebraska Medical Center, Omaha, NE; 4 Division of Gastroenterology and Hepatology, Department of Internal Medicine, University of Nebraska Medical Center, Omaha, NE

## Abstract

Turmeric is a common herbal supplement used for its possible anti-inflammatory and other properties. It is marketed as safe with few reports of major adverse effects directly related to oral supplementation. We report a case of turmeric supplement-induced liver injury in a 49-year-old woman admitted with elevated aspartate aminotransferase and alanine aminotransferase with no history of liver disease or alcohol use disorder. Thus, this case re-emphasizes the importance of evaluating herbal and dietary supplements as potential drug-induced liver injury causes.

## INTRODUCTION

Idiosyncratic drug-induced liver injury (DILI) is estimated to occur in up to 1 of 10,000 patients who take medications; however, diagnosis is challenging owing to the numerous alternative etiologies that must be excluded.^1^ In the United States, DILI has been implicated in 13% of acute liver failure cases, with herbal/dietary supplements accounting for 9%.^1,2^

Turmeric is an herbal and dietary product, one of the most used supplements in the United States.^3^ Reportedly, it offers anti-inflammatory benefits while demonstrating safety with minimal adverse effects.^4–6^ The major active ingredient in turmeric is curcumin, with some formulations adding piperine.^7^ We present a case of DILI attributed to a turmeric supplement, with characterization, quantitation, and complete recovery on discontinuation of the product.

## CASE REPORT

A 49-year-old White woman with a history of ocular hypertension, polycystic ovarian syndrome, and cholecystectomy presented with fatigue, nausea, jaundice, and mild left upper quadrant abdominal pain with progressively darkened urine for 1 week. She denied any fever, changes in bowel habits, new skin lesions, significant alcohol consumption, and illicit or recreational drug use. She denied any medication changes, except for starting a turmeric supplement approximately 1.5 months before, which she discontinued a day before admission. She reported taking 2 capsules daily. She endorsed no recent travel in the 2 months before presentation. Her other medications included *Lactobacillus rhamnosus*, vitamin C, latanoprost ophthalmic solution, timolol ophthalmic solution, and levonorgestrel intrauterine device. She also reported a headache that lasted for 1 day the week before presentation, for which she took 1 to 2 tablets of a combination aspirin, caffeine, and acetaminophen pill.

Her vitals on admission were unremarkable. She was in no acute distress but was jaundiced. No hepatosplenomegaly, ascites, encephalopathy, nor other stigmata of chronic liver disease were noted. Her initial laboratory results were unremarkable, except for her liver chemistries (Table 1). Her R-score was calculated at 42, consistent with a hepatocellular pattern of liver injury. Evaluation for viral hepatitis, alcohol-induced liver injury, and ischemic hepatopathy was unremarkable (Table 1). An abdominal ultrasound was unremarkable, and a magnetic resonance cholangiopancreaticogram revealed no additional findings.

**Table 1.** Day of presentation vs previous laboratory diagnostics

Component (reference ranges)	Day of presentation	5 yr before (most recent before admission)
White blood cell (4,000–11,000 cells/mm^3^)	4,700 cells/mm^3^	
Hemoglobin (11–15.1 g/dL)	14.7 g/dL	
Hematocrit (33.1%–44.5%)	46%	
Platelet (150,000–400,000 cells/mm^3^)	226,000 cells/mm^3^	
Blood urea nitrogen (6–20 mg/dL)	13 mg/dL	
Creatinine (0.44–1.03 mg/dL)	0.58 mg/dL	
Alkaline phosphatase (32–91 U/L)	138 U/L	63 U/L
Aspartate aminotransferase (15–41 U/L)	1,894 U/L	19 U/L
Alanine aminotransferase (7–52 U/L)	3,289 U/L	19 U/L
Total bilirubin (0.3–1 mg/dL)	7.1 mg/dL	0.5 mg/dL
International normalized ratio (0.9–1.1)	1	
Albumin (3.5–5.1 g/dL)	4.4 g/dL	
Thyroid stimulating hormone (0.4–4.3 μIU/mL)	1.527 μIU/mL	
Free T4 (0.6–1.6 ng/dL)	1 ng/dL	
Hepatitis A, B, C, E	Negative	
Cytomegalovirus	Negative	
Parvovirus	Negative	
Herpes simplex virus 1 and 2	Negative	
HIV 1 and 2	Negative	
Human papilloma virus	Negative	
Epstein-Barr virus	Consistent with previous exposures	
Urine drug screen	Negative	
Blood alcohol (<10 mg/dL)	<10 mg/dL	
Acetaminophen level (0–30 μg/mL)	<10 μg/mL	
Salicylate level (0–19.9 mg/dL)	<2.5 mg/dL	
Serum immunoglobulin G (610–1,616 mg/dL)	1,300 mg/dL	
Antinuclear antibody Hep-2 substrate titer	1:320 with a speckled pattern	
Anti-smooth muscle antibody titers	Negative	
Anti-liver-kidney-microsomal antibodies	Negative	
Anti-mitochondrial antibodies	Negative	

**Figure 1.**
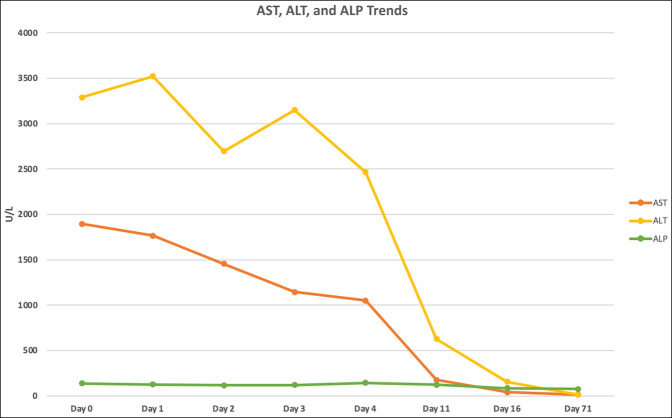
Trend of aspartate aminotransferase (AST), alanine aminotransferase (ALT), and alkaline phosphatase (ALP).

**Figure 2.**
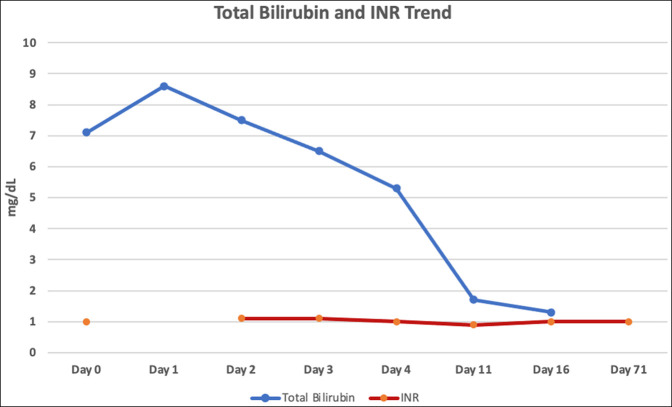
Trend of total bilirubin and international normalized ratio (INR).

A liver biopsy was not performed because her liver chemistries on day 3 of hospitalization showed an improvement of >45% in her aspartate aminotransferase and alanine aminotransferase. On discharge, she was instructed not to restart her turmeric supplement; however, all other medications before admission were restarted. A follow-up visit 71 days after the initial presentation demonstrated complete resolution of her jaundice and liver chemistries (Figures 1 and 2). The patient was not rechallenged with her turmeric supplement. No immunosuppressive therapies were initiated at any point during her treatment.

The turmeric supplement was reviewed, and the label reported the following ingredients: turmeric 1,000 mg (950 mg curcuminoids), black pepper 10 mg (9 mg piperine), vegetarian capsule (hypromellose), microcrystalline cellulose, vegetable magnesium stearate, and silicon dioxide. The sample was sent for mass spectrometry analysis, and the curcumin concentrations are listed in Table 2.

**Table 2.** Curcumin concentrations (N = 5 capsules)

Curcumin component	Concentrations (mg) Mean ± SD
Curcumin	467.5 ± 135.8
Demethoxycurcumin	72.6 ± 18.37
Bisdemethoxycurcumin	9.5 ± 2.5

## DISCUSSION

Recently, there has been an increasing amount of in vivo data to support turmeric's anti-inflammatory, antioxidant, antiproliferative, and antineoplastic properties.^4^ However, data from 8 US centers in the Drug-Induced Liver Injury Network revealed that between 2004 and 2013, 15.5% (130/839) of hepatotoxicity cases were attributed to dietary and herbal supplements.^8^ Of the 130 cases, turmeric was associated with 32 of the single-ingredient products.^9,10^

Turmeric products are listed by their curcuminoid content, typically exhibit poor bioavailability, and have an approximate half-life of 6–7 hours.^5^ Curcumin's bioavailability may be improved with formulation modifications including formulating it concomitantly with the active constituent of black pepper or piperine.^11^ Some trials have shown that turmeric is well-tolerated, with a suggested target dose of 4,000–8,000 mg.^4^

This report highlights a case of DILI attributed to a turmeric supplement, formulated with piperine with a latency period of approximately 1.5 months. The patient's R-score of 42 was consistent with a hepatocellular pattern of liver injury. In addition, because DILI is a diagnosis of exclusion, an extensive workup was completed and was unremarkable. Viral serologies were negative or indicative of previous exposure, there was no evidence of hepatic or biliary pathology on magnetic resonance cholangiopancreaticogram, and autoimmune serologies did not strongly suggest autoimmune hepatitis. Unlike other cases of drug-induced autoimmune hepatitis attributed to a turmeric supplement, improvement on discontinuation of the supplement in the absence of immunosuppressive treatment suggests against autoimmune hepatitis.^13,14^ Based on the temporal relationship and improvement of the liver chemistries on discontinuation of the turmeric product, the diagnosis was consistent with turmeric DILI. An updated Roussel Uclaf Causality Assessment Method score was calculated to be 7, indicating a probable association (scores ≥9 indicate a highly probable association).^12^

Previous case reports have not characterized the actual product attributed to DILI.^13,14^ In this report, the product was identified, its label was reviewed, and it clarified the absence of other hepatotoxic ingredients. The curcumin components were less than the label-reported amount. Thus, there is concern that potentially lower doses of curcumin in combination with piperine may not be well-tolerated. More recently, a study assessed possible alleles that may be a risk factor of liver injury from herbal components; however, it acknowledged that further studies for the potential synergistic hepatotoxicity associated with turmeric and piperine are needed to elucidate this mechanism of liver injury.^15^ From the case series, liver injury was hepatocellular with a latency period of 1–4 months, which was consistent with this case report.

In conclusion, this report demonstrates turmeric-associated DILI and the importance of recognizing the supplement as a potential cause of hepatic injury. It further emphasizes the importance of detailed medication history and reconciliation, which includes herbal and dietary supplements. It also demonstrates the significance of patient medication counseling and education, so patients know the potential risks and adverse effects of over-the-counter medications. Finally, an interprofessional inquiry into patient's utilization of natural and complementary medicines can assist providers in obtaining essential information regarding possible DILI culprits and potentially preventing further exposure, which could lead to significant adverse effects.

## DISCLOSURES

Author contributions: SW Sunagawa wrote and revised the manuscript and is the article guarantor. C. Houlihan assisted with writing and revising the manuscript. DJ Murry completed the mass spectrometry methodology and analysis. B. Reynolds and S. Kjeregtroen supervised and mentored the efforts of the other study members. N. Khoury revised the manuscript.

Financial disclosure: None to report.

Informed consent was obtained for this case report.

## References

[1] Chalasani N Fontana RJ Bonkovsky HL . Causes, clinical features, and outcomes from a prospective study of drug-induced liver injury in the United States. Gastroenterology. 2008;135(6):1924–34, 1934.e1–4. 18955056 10.1053/j.gastro.2008.09.011 PMC3654244

[2] Ostapowicz G Fontana RJ Schiodt FV . Results of a prospective study of acute liver failure at 17 tertiary care centers in the United States. Ann Intern Med. 2002;17(12):947–54. 10.7326/0003-4819-137-12-200212170-00007 12484709

[3] Smith T Gillespie M Eckl V . Herbal supplemental sales in US increase by 9.4% in 2018 record growth driven by sales of CBD, mushrooms, and immune-health products. Herbalgram. 2019;123:62–73.

[4] Gupta SC Patchva S Aggarwal BB. Therapeutic roles of curcumin: Lessons learned from clinical trials. AAPS J. 2013;15(1):195–218. 23143785 10.1208/s12248-012-9432-8 PMC3535097

[5] Lao CD Ruffin MT Normolle D . Dose escalation of a curcuminoid formulation. BMC Complement Alternat Med. 2006;6:article 10. 10.1186/1472-6882-6-10 PMC1434783 16545122

[6] Kurien BT Danda D Schofield FH. Therapeutic potential of curcumin and curcumin analogues in rheumatology. Int J Rheum Dis. 2015;18(6):591–3. 26301972 10.1111/1756-185X.12753 PMC7301892

[7] Qin S Huang L Gong J . Meta-analysis of randomized controlled trials of 4 weeks or longer suggest that curcumin may afford some protection against oxidative stress. Nutr Res. 2018;60:1–12. 30527253 10.1016/j.nutres.2018.08.003

[8] Navarro VJ Barnhart H Bonkovsky HL . Liver injury from herbals and dietary supplements in the US Drug-Induced Liver Injury Network. Hepatology. 2014;60(4):1399–408. 25043597 10.1002/hep.27317 PMC4293199

[9] Jager R Lowery RP Calvanese AV . Comparative absorption of curcumin formulations. Nutr J. 2014;13:11. 24461029 10.1186/1475-2891-13-11 PMC3918227

[10] Purpura M Lowery RP Wilson JM . Analysis of different innovative formulations of curcumin for improved relative oral bioavailability in human subjects. Eur J Nutr. 2018;57(3):929–38. 28204880 10.1007/s00394-016-1376-9 PMC5861163

[11] Tabanelli R Brogi S Calderone V. Improving curcumin bioavailability: Current strategies and future perspectives. Pharmaceutics. 2021;13(10):1715. 34684008 10.3390/pharmaceutics13101715 PMC8540263

[12] Danan G Teschke R. RUCAM in drug and herb induced liver injury: The update. Int J Mol Sci. 2015;17(1):14. 26712744 10.3390/ijms17010014 PMC4730261

[13] Lukefahr AL McEvoy S Alfafara C . Drug-induced autoimmune hepatitis associated with turmeric dietary supplement use. DMJ Case Rep. 2018;2018:bcr-2018-224611. 10.1136/bcr-2018-224611 PMC6144106 30206065

[14] Lee BS Bhatia T Chaya CT . Autoimmune hepatitis associated with turmeric consumption. ACG Case Rep J. 2020;7(3):e00320. 32337301 10.14309/crj.0000000000000320 PMC7162126

[15] Halegoua-DeMarzio D Navarro V Ahmad J . Liver injury associated with turmeric—A growing problem: Ten cases from the Drug-Induced Liver Injury Network [DILIN]. Am J Med. 2022. 10.1016/j.amjmed.2022.09.026 PMC9892270 36252717

